# TGx-DDI, a Transcriptomic Biomarker for Genotoxicity Hazard Assessment of Pharmaceuticals and Environmental Chemicals

**DOI:** 10.3389/fdata.2019.00036

**Published:** 2019-10-08

**Authors:** Heng-Hong Li, Carole L. Yauk, Renxiang Chen, Daniel R. Hyduke, Andrew Williams, Roland Frötschl, Heidrun Ellinger-Ziegelbauer, Syril Pettit, Jiri Aubrecht, Albert J. Fornace

**Affiliations:** ^1^Department of Oncology, Department of Biochemistry and Molecular & Cellular Biology, Georgetown University Medical Center, Washington, DC, United States; ^2^Environmental Health Science and Research Bureau, Health Canada, Ottawa, ON, Canada; ^3^Amelia Technologies LLC, Rockville, MD, United States; ^4^Federal Institute for Drugs and Medical Devices, Bonn, Germany; ^5^Investigational Toxicology, Bayer AG, Pharmaceuticals, Leverkusen, Germany; ^6^Health and Environmental Sciences Institute, Washington, DC, United States

**Keywords:** TGx-DDI, toxicogenomics, genotoxicity, biomarker, high-throughput (HT) approaches

## Abstract

Genotoxicity testing is an essential component of the safety assessment paradigm required by regulatory agencies world-wide for analysis of drug candidates, and environmental and industrial chemicals. Current genotoxicity testing batteries feature a high incidence of irrelevant positive findings—particularly for *in vitro* chromosomal damage (CD) assays. The risk management of compounds with positive *in vitro* findings is a major challenge and requires complex, time consuming, and costly follow-up strategies including animal testing. Thus, regulators are urgently in need of new testing approaches to meet legislated mandates. Using machine learning, we identified a set of transcripts that responds predictably to DNA-damage in human cells that we refer to as the TGx-DDI biomarker, which was originally referred to as TGx-28.65. We proposed to use this biomarker in conjunction with current genotoxicity testing batteries to differentiate compounds with irrelevant “false” positive findings in the *in vitro* CD assays from true DNA damaging agents (i.e., for de-risking agents that are clastogenic *in vitro* but not *in vivo*). We validated the performance of the TGx-DDI biomarker to identify true DNA damaging agents, assessed intra- and inter- laboratory reproducibility, and cross-platform performance. Recently, to augment the application of this biomarker, we developed a high-throughput cell-based genotoxicity testing system using the NanoString nCounter® technology. Here, we review the status of TGx-DDI development, its integration in the genotoxicity testing paradigm, and progress to date in its qualification at the US Food and Drug Administration (FDA) as a drug development tool. If successfully validated and implemented, the TGx-DDI biomarker assay is expected to significantly augment the current strategy for the assessment of genotoxic hazards for drugs and chemicals.

## Introduction

Genetic alterations are an important hallmark of cancer, and DNA damage (genotoxic stress) is a central driver of carcinogenesis. Therefore, the assessment of a chemical's potential to induce gene mutations and/or chromosomal damage has been formalized in various genotoxicity testing batteries that are widely accepted and provide insight into the carcinogenic potential of drug candidates, cosmetics, food additives, environmental, and industrial chemicals. In general, genotoxicity testing batteries consist of standardized *in vitro* and *in vivo* assays, and have been successful in protecting human health through identification of genotoxic and carcinogenic exposures (Snyder and Green, 2001).

Although the mechanistic association between genetic damage and cancer is well-understood, the correlation between genotoxicity, the potential of chemicals to induce primary DNA damage, and carcinogenic potential in animals or humans is complicated by alternative non-genotoxic mechanisms of carcinogenesis and the experimental limitations of *in vitro* genotoxicity assays. In fact, many compounds testing positive in genetic toxicology batteries, particularly with *in vitro* chromosomal damage (CD) assays, do not represent an appreciable carcinogenic risk to humans (Snyder and Green, 2001). *In vitro* CD assays are designed to detect structural chromosomal abnormalities induced by the test compounds. The widely used CD assays include the chromosomal aberration assay, and micronucleus assay. All mammalian cell genotoxicity tests have low specificity of below 45%, which become more severe when two or three tests are combined. For example, it was reported that 75–95% of non-carcinogens gave positive (i.e., false positive) results in at least one standard *in vitro* genotoxicity test (Kirkland et al., 2005). In drug development, a larger percent of new lead molecules are expected to yield potentially irrelevant positive *in vitro* genotoxicity findings. Since assessing carcinogenicity in animal studies during the early stages of drug development is often not feasible, potentially safe drug candidates may be excluded from the development pipeline. Therefore, the differentiation of true positives from irrelevant positives in the *in vitro* tests, such as the *in vitro* CD assays, is crucial for the initial assessment of human risk. This concept is also reflected in the current Food and Drug Administration (FDA) guidance for industry (https://www.fda.gov/downloads/Drugs/GuidanceComplianceRegulatoryInformation/Guidances/UCM079257.pdf).

Currently, there are two approaches used to address the risk and relevance of positive *in vitro* CD assays. The first approach relies on characterizing the risk and relevance of the positive results by conducting additional *in vitro* and *in vivo* studies that use similar DNA damage sensing endpoints to explore a limited number of mechanisms, such as γH2AX, a marker for DNA double strand breaks (Redon et al., 2011), phospho-Histone H3 (a marker for mitotic cells) (Ando et al., 2014; Bryce et al., 2014), and general apoptosis assays. These experimental follow-up strategies often have uncertain outcomes, are costly, laborious, and can lead to discontinuation of drug development and/or significant delays in the introduction of new medications to patients. The second approach, developed by the International Conference on Harmonization (ICH), relies on two *in vivo* tests as an alternative to the *in vitro* CD assays (https://www.fda.gov/downloads/drugs/guidances/ucm074931.pdf). Although this approach increases human relevance via *in vivo* testing, the DNA damage endpoints in the *in vivo* studies are inherently difficult to assess and do not increase our understanding of the genotoxic mechanisms responsible for *in vitro* irrelevant positive results. Furthermore, in most cases this requires additional animal studies, unless the evaluation of *in vivo* endpoints is combined with, or included in, other toxicity studies. Often an important arbiter for conflicting *in vitro* results involves expensive and time-consuming *in vivo* carcinogenesis studies. Therefore, there is an urgent need for mechanistic tools that do not rely on animal use to aid in the interpretation and risk assessment of isolated positive findings from *in vitro* genotoxicity assays.

Monitoring transcriptional changes as an indicator of the DNA damage response has been explored as a tool for assessing genotoxicity (Amundson et al., 2005; Li et al., 2007). DNA damage responses initiate from damage sensing and signaling mediated by sensor molecules such as the MRN complex, Rad17, MDC1, 53BP1, and BRCA1 (Bakkenist and Kastan, 2004; O'Driscoll and Jeggo, 2006), and signaling kinases including ATM, ATR, DNA-PK, Chk1, and Chk2 (McGowan and Russell, 2004). Activation of this elaborate DNA damage response network induces transcription of DNA-damage responsive genes that are involved in DNA damage repair, cell cycle control, apoptosis, and metabolic regulation. Many of these responses are dependent on the p53 transcription factor, but additional genotoxic-stress induced transcriptional responses exist even in p53-deficient cells (Amundson et al., 2005). Despite the complexity of biological responses to xenobiotics, the key transcriptional factors activated in DNA damage response can be distinguished from those induced by non-genotoxic stress (Amundson et al., 2005; Jennings et al., 2013); the latter fundamental feature enables the identification of genotoxicity-induced transcriptomic modulations that can be distinguished from those triggered by other stresses.

An approach applying transcriptomics to identify DNA damage inducing agents offers the advantage of informing whether an intact DNA damage response has been initiated by a toxicant that is consistent with the transcript profiles of known genotoxic carcinogens and distinct from non-genotoxic mechanisms. These transcriptomic biomarkers detect DNA damage as induction of a genotoxic stress response that is measured as transcriptional activation of early immediate DNA damage response genes, reflecting primary DNA damage. These cellular responses happen before activation of apoptosis or cell death, the latter of which might also occur following prolonged compound treatment (Ellinger-Ziegelbauer et al., 2009a). In contrast, mutations or chromosome damage occurring as a consequence of repair of damaged DNA are what is detected using the standard genetic toxicology assay battery. Thus, cellular stressor not primarily attacking DNA can induce chromosome damage that is detected using the conventional *in vitro* test systems. In addition to providing insight into the relevance and mechanisms associated with observed *in vitro* genotoxicity in conventional tests, it is envisioned that transcriptomic approaches could provide a tool for high-throughput genotoxicity screens for not only pharmaceutical compounds but also environmental and occupational toxicants. Below we describe the development, validation, and application of the TGx-DDI transcriptomic biomarker for these purposes.

## Development of the TGx-DDI Transcriptomic Biomarker

**Figure 1:**
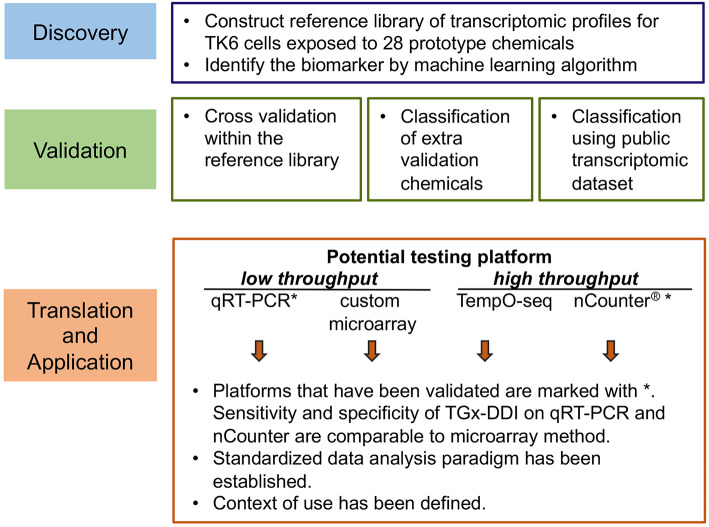
Overview of the development phases of the transcriptomic genotoxicity biomarker TGx-DDI.

An *in vitro* transcriptome-based approach was used to develop a biomarker to assess genotoxicity and provide insights into mode of action (Li et al., 2015). The process involved in the development of the biomarker, including identification, testing and validation, as well as relevant case studies to explore context of use, are reviewed below. A schematic chart summarizing these stages is shown in Figure 1.

### Identification of the TGx-DDI Biomarker

#### (1) Cell System and Model Agents

Transcriptome perturbations were determined using the TK6 cell line as (a) it is a well-characterized cell line that is routinely used in genotoxicity testing, (b) it has proficient p53 signaling pathways, and (c) it responded in an expected manner in previous stress response studies (Akerman et al., 2004; Dickinson et al., 2004; Hu et al., 2004; Islaih et al., 2004; Amundson et al., 2005). Unlike human cancer cell lines, the TK6 line was established by spontaneous immortalization of primary human lymphoid cells in culture, and probably doesn't have the myriad of genetic alterations typically seen in lines derived from human cancers (Skopek et al., 1978; Liber and Thilly, 1982).

The TGx-DDI biomarker (TGx = toxicogenomic; DDI = DNA damage inducing) was identified using a dataset comprised of transcriptomic profiles of TK6 cells exposed to 28 model agents with well-characterized modes of action (Kirkland et al., 2005; Bryce et al., 2014). The agents in this training set represent a wide range of DDI mechanisms and include DNA alkylating agents, DNA strand breaking agents, topoisomerase inhibitors, and nucleotide antimetabolites, and non-DDI agents such as endoplasmic reticulum (ER) stress agents, energy metabolism inhibitors, HDAC inhibitors, heat shock, and oxidative agents including heavy metals. The biomarker is specific to the identification of DNA damage; thus, it is important to note that aneugens are classified as non-DDI in this analysis as they operate via interaction with the mitotic spindle.

#### (2) Study Design Considerations for Transcriptional Profiling

Several study design parameters are critical for the development and use of transcriptomic biomarkers of DNA damage stress response. Complex cellular stress responses following treatment with chemicals are time- and dose-dependent. However, an experimental design that includes characterizing dose responses over a time course is not technically feasible across a large set of agents using conventional global transcriptomic approaches (i.e., DNA microarrays). Moreover, selection of single concentrations and time points facilitates extraction of relevant gene sets. Thus, the initial experimental design used a single dose and time point post-exposure for transcriptomic profiling.

A 4 h post-exposure time point was selected for the development of the biomarker (Fornace et al., 1988; Amundson et al., 2005; Hyduke et al., 2011) as early gene expression changes reflect initial damage rather than subsequent cellular processes such as cell lethality events (Ellinger-Ziegelbauer et al., 2009b). In addition, 4 h is sufficient time to allow for accumulation of typical DNA damage response induced transcripts (Fornace et al., 1988, 1989).

To identify an appropriate concentration for each model agent and make sure that a sufficient cellular transcriptional response was achieved, mRNA levels of three well-characterized stress response genes (*GADD45A, ATF3*, and *CDKN1A*) were assessed. The concentration of each agent employed for transcriptomics was the one that produced a robust increase of mRNA levels of one or more of these stress genes in this preliminary experiment. When more than one concentration met the criteria and behaved similarly, the lower concentration was selected. For agents included in the development of TGx-DDI biomarker (i.e., learning set), the concentration ranges tested, and final concentrations chosen for the microarray analyses are included in Table 1. The concentrations selected showed no appreciable cytotoxicity at 4 h and the cell viability at 24 h post-treatment showed only moderate effects on viability for most agents. It should also be noted that the use of high-throughput approaches, such as the Nanostring approach discussed in this review, obviates the need for dose optimization, because a wide dose range is now feasible.

**Table 1:** Agents used in the development of the TGx-DDI transcriptomic biomarker (See also Li et al., 2015).

**Categories**	**Compound names**	**Solvent**	**Dose range**	**Conc. for array**
Alkylating agents	Cisplatin Methyl methane sulfonate (MMS)	0.9% NaCl H_2_O	10 ~ 80 μM 20 ~ 200 μg/ml	80 μM 100 μg/ml
Topoisomerase I inhibitors	Camptothecin	DMSO	62.5 ~ 500 nM	125 nM
Topoisomerase II inhibitors	Etoposide	DMSO	50 ~ 400 nM	200 nM
RNA/DNA antimetabolites	5-fluorouracil (5-FU) Methotrexate	DMSO DMSO	6.25 ~ 50 μg/ml 0.05 ~ 1 mM	25 μg/ml 100 μM
DNA antimetabolites	Arabinofuranosyl cytidine (AraC) Hydroxyurea	H_2_O H_2_O	12.5~ 50 μM 0.25 ~ 1 mM	50 μM 0.5 mM
Causing DNA strand break by other mechanisms	γ-rays Bleomycin Hydrogen peroxide	N/A^*^ H_2_O N/A^*^	4 Gy 5 ~ 40 μg/ml 20 ~ 80 μM	4 Gy 10 μg/ml 80 μM
Antimitotic agents	Colchicine Docetaxel Paclitaxel Vinblastin	Ethanol DMSO DMSO DMSO	62.5 ~ 1000 ng/ml 25 ~ 100 nM 12.5 ~ 200 nM 50 ~ 800 ng/ml	250 ng/ml 50 nM 50 nM 200 ng/ml
Histone modification inhibitors	Trichostatin A (TSA) Apicidin HC toxin Oxamflatin	DMSO DMSO Methanol DMSO	5 ~ 80 ng/ml 0.25 ~ 4 μg/ml 5 ~ 80 ng/ml 0.25 ~ 4 μM	20 ng/ml 1 μg/ml 20 ng/ml 1 μM
Endoplasmic reticulum modulator	Tunicamysin Thapsigargin	Methanol Ethanol	1.25 ~ 10 μg/ml 62.5 ~ 500 nM	2.5 μg/ml 250 nM
Glycolysis inhibitor	2-deoxy-D-glucose (2-DG)	H_2_O	0.16 ~ 20 μM	20 μM
Energy metabolism inhibitor (uncoupling agent)	Antimycin A	Ethanol	25 ~ 200 μM	100 μM
Heavy metals	Cadmium chloride Potassium chromate (VI) Sodium arsenite	H_2_O H_2_O H_2_O	50 ~ 800 μM 25 ~ 400 μM 10 ~ 90 μM	50 μM 100 μM 30 μM
Other stresses	Heat shock ethanol	N/A^*^ N/A^*^	47°C 2%, 4%	47°C 2%, 4%

The first column indicates the mechanisms of action as described in the text. The last three columns list the solvent used, the dose range tested, and the final dose selected for transcriptomic analyses.

* N/A, not applicable.

#### (3) Data Analysis Pipeline Used to Identify the TGx-DDI Biomarker

**Figure 2:**
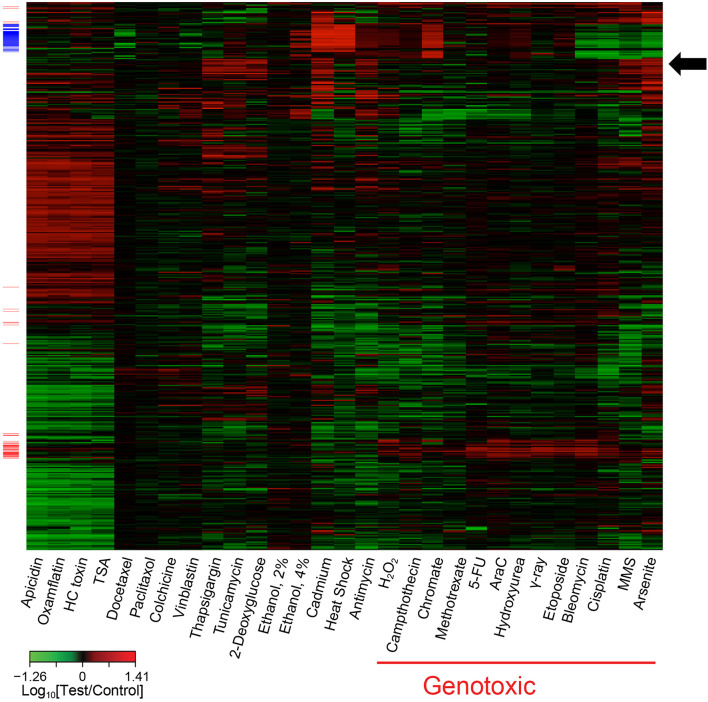
Heatmap of transcriptome profiling data illustrating co-expressed sets of genes associated with various toxicants, such as genotoxic agents (red side bars). Some agents also exhibit obvious pleiotropy; e.g., chromate induced expression of genes associated with genotoxic agents (red side bars) and genes associated with cadmium and heat shock (blue side bars). This heatmap contains 1,628 genes that were significantly (*P* < 0.01; *t*-test) perturbed at least 1.7-fold, relative to the control, by at least one stress agent. The genes in the heatmap were organized by hierarchical clustering with complete linkage based on their error-weighted Pearson distances. The genotoxic (red side bars) and cadmium and heat shock (blue side bars) signatures were identified using coupled two-way clustering. The arrow highlights a cluster of genes responding to ER stress agents thapsigargin and tunicamycin as well as certain other agents.

The global transcriptomic responses to treatment with the 28 model agents at the selected concentrations were measured using Agilent human whole genome oligonucleotide arrays. The individual gene expression profiles were then compiled in a database for further analysis. To limit noise in the data and enable interrogation of the database of gene expression profiles for gene signatures, genes that were significantly perturbed in three or more of the treatment conditions were selected. Although the gene expression profiles of the individual agents in the reference database displayed significant diversity, subsets of genes often formed clusters that were consistent with their mode of action. Visualization of the expression profiles highlighted the pleiotropic nature of these agents (i.e., gene expression profiles consisting of more than one defined gene cluster) (Figure 2). Further analysis using the nearest shrunken centroids (NSC) method led to the identification of a biomarker capable of identifying DDI agents. In brief, the standardized centroid for each class was computed, where the standardized centroid was the mean expression level for each gene in a class divided by its within-class standard deviation. The standard centroid for each class was shrunken toward the overall centroid to produce the NSC. The method employed a shrinkage parameter to control the number of features used to construct the classifier. With a shrinkage threshold of 2.2, a 65-transcript panel was identified. The prediction for DDI probability reached 100% accuracy as assessed by 10-fold cross validation for the agents in this reference database. This transcriptomic biomarker was initially referred to as TGx-28.65 (reflecting 28 model agents in the training set, and 65 probes used in the signature). The 65 transcripts on the microarray probe design were later aligned to 64 genes as two of the transcripts were annotated to a single gene. To avoid confusion relating to the number of genes included in this biomarker, the biomarker was later renamed TGx-DDI. For a complete list of the genes in the TGx-DDI biomarker refer to Table 2 in Li et al. (2015). Pathway analysis of the biomarker genes revealed that p53 signaling and cell cycle regulation are the top two enriched pathways in this gene set.

### Validation of TGx-DDI—A Robust Biomarker for Genotoxicity

To evaluate the sensitivity and specificity of the TGx-DDI biomarker for the assessment of genotoxic hazard associated with DNA damage, 45 chemicals were tested, which covered five classes of distinct genotoxic mechanisms and phenotypes (Kirkland et al., 2005; Li et al., 2017) (Table 2). These compounds were selected based on adjudication of published data that included expert input from members of the FDA biomarker qualification team, and information from databases including the carcinogenicity potency database (https://toxnet.nlm.nih.gov/newtoxnet/cpdb.htm) and genotox databases also available via TOXNET (https://toxnet.nlm.nih.gov/). For chemicals in the validation study, initial cell viability assessment guided the selection of concentrations analyzed in dose setting experiments using the three gene stress response panel described above. Transcriptomic profiling using DNA microarrays was performed on the single concentration selected for each chemical following the described selection process above.

**Table 2:** Summary of the TGx-DDI validation results on 48 external test agents.

**Class^**&**^**	**Agents**	**TGx**-**DDI**				**Genotoxicity test**	
		PA^*^	**2DC^**#**^**	**PCA^**@**^**	**Overall**	**CD^**%**^**	**AMES**
Class 1	N-Ethyl-N-Nitrosourea (ENU)	+	+	+	+	+	+
	Mitomycin (MMC)	+	+	+	+	+	+
	Ethyl methanesulfonate (EMS)	+	+	+	+	+	+
	Belomycin	+	+	+	+	+	+
	Nitrogen mustard	+	+	+	+	+	+
	Chlorambucil	+	+	+	+	+	+
	Busulfan	–	+	–	+	+	+
	Isopropyl methanesulfonate	+	+	+	+	+	+
	Hydroquinone	–	+	+/–	+	+	+
Class 2	2A: Topoisomerase inhibitors						
	Doxorubicin	+	+	+	+	+	+
	Genistein	+	+	+	+	+	–
	Topotecan	+	+	+	+	+	+
	Norfloxacin	–	–	–	–	+	–
	Ciprofloxacin	+/–	+	+	+	+	–
	2B: Antimetabolites						
	5-Fluorouracil (5-FU)	+	+	+	+	+	+
	Thioguanine (6-TG)	+	+	+	+	+	–
	Thiopurine (6-MP)	–	–	–	–	+	–
	Azidothymidine (AZT)	–	–	–	–	+	–
	5-azacytidine (5-AzaC)	–	–	–	–	+	+
Class 3	3A: Kinase inhibitors						
	Dasatinib	+	+	+	+	+	–
	Imatinib mesylate	–	–	–	–	+	–
	Sorafenib	–	–	–	–	+	–
	3B: Additional compounds						
	Benomyl	–	–	–	–	+	–
	Diethylstilbestrol	–	+	+	+	+	–
	Nocodazole	–	–	–	–	+	–
Class 4	4A: Kinase inhibitors						
	Sunitinib maleate	–	–	–	–	–	–
	Gefinitib	–	–	–	–	–	–
	Progesterone	–	–	–	–	–	–
	Diethanolamine	–	–	–	–	–	–
	Melamine	–	–	–	–	–	–
	4C: Antibiotics						
	Ampicillin	–	–	–	–	–	–
	Erythromycin Stearate	–	–	–	–	–	–
	4D: Others						
	D-Mannitol	–	–	–	–	–	–
	n-Butyl Chloride	–	–	–	–	–	–
	3-Nitropropionic acid	–	–	–	–	–	–
	Methyl Carbamate	+	+/–	+	+	–	–
Class 5	Phenobarbital	–	–	–	–	+	–
	Esomeprazole	–	–	–	–	+	–
	Donepezil	–	–	–	–	+	–
	Cyclohexamide	–	–	–	–	+	–
	2,4 Dinitrophenol (2,4 DNP)	–	–	–	–	+	–
	Olmesartan	–	–	–	–	+	–
	Exemastan	+	+	+	+	+	–
	Rabeprazole-NA	–	–	–	–	+	–
	Rotigotin	–	–	–	–	+	–
	Dexamethasone	–	–	–	–	+	–
	Caffeine	–	–	–	–	+	–
	Staurosporine	–	–	–	–	+	–

& Chemical classes: Class 1, DDI agents that interact directly with DNA; Class 2, DDI agents that interact indirectly with DNA; Class 3, Kinase inhibitors and other compounds that may interact indirectly with DNA, with positive CD findings; Class 4, Non-genotoxic agents; Class 5, Agents with irrelevant positives for in vitro assays.

* PA, Probability Analysis.

# 2DC, 2-Dimensional Clustering.

@ PCA, Principle Component Analysis.

% CD, Chromosomal Damage Assay.

*Validated chemicals are listed by classes. The column “TGx-DDI” shows results from the three analysis methods and the “Overall” prediction (positive in any one = +; negative in all three = –). The column “Genotoxicity tests” includes the results of CD and the Ames assay documented elsewhere*.

#### (1) Data Analysis for Test Compound Classification

A paradigm consisting of three data analysis methods, (a) two-dimensional hierarchical clustering (2DC), (b) principal component analysis (PCA), and (c) NSC-posterior probability analysis (PA), was applied to the profiling data of the 45 test agents to classify each chemical as DDI or non-DDI. A chemical was classified as DDI if it gave a positive call in any one of these three prediction analyses. Non-DDI classification was assigned only to chemicals that did not meet any of these criteria (Li et al., 2017). This three-pronged classification paradigm decreases the number of false negatives, which is important for safety assessment. In general, when analyzing transcriptomic data, it has been recommended to use more than one analysis (Alvo et al., 2010).

When the three-pronged analysis was applied to our training set, the 2DC analysis that relies on the Euclidian distance and average linkage clearly differentiated the DDI and non-DDI agents into two main clusters. In the PCA, the first principal component separated all DDI and non-DDI agents (Williams et al., 2015; Li et al., 2017). Finally, the NSC-PA that relies on centroids with probability cutoff (P > 0.9) differentiated the DDI class from the non-DDI class.

To make this TGx-DDI analytical pipeline conveniently accessible to the public, a user-friendly online software tool has been developed (https://manticore.niehs.nih.gov/tgxddi/tool) (Jackson et al., 2017).

#### (2) Conclusions From Validation Studies

**Figure 3:**
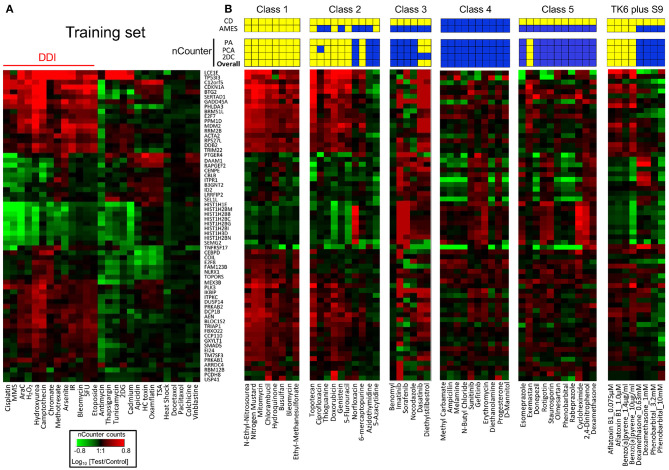
Performance of TGx-DDI with nCounter® analysis system. **(A)** Heatmap of Nanostring expression analysis using previously tested chemicals; all chemicals were classified as DDI or non-DDI using the same approach used in the DNA microarray analysis. IR designates ionizing radiation. **(B)** Thirty-eight chemicals were grouped based on mechanistic properties (Table 1); four chemicals that require metabolic activation were evaluated at different concentrations; heatmaps are shown for nCounter® results for each class and prediction results are displayed above. Three methods were used to predict DDI positive (yellow), and the overall prediction (bottom row) is based on positive results with any of these three methods. Published results with CD and Ames assays are shown in the top 2 rows. Yellow and blue indicate positive and negative findings, respectively.

Table 2 and Figure 3 summarize the results of the TGx-DDI validation set of 45 chemicals that represented five mechanistic classes. Overall, the three-pronged analytical approach yielded the expected classifications, with few exceptions. All agents in class 1, consisting of alkylating agents, were classified as DDI. With the exception of one agent (methyl carbamate), all chemicals in class 4 were classified as non-DDI. All but one agent (exemastan) in class 5 were classified as non-DDI. Since class 5 consisted of agents with known irrelevant positive findings in the *in vitro* CD assays, these results indicate that TGx-DDI has a potential to de-risk these findings. Agents in class 3, kinase inhibitors and other compounds with positive CD results, which may interact indirectly with DNA, were classified as non-DDI with two exceptions: dasatinib and diethylstilbestrol. Class 2A agents, all mammalian topoisomerase inhibitors, were classified as DDI. In contrast, norfloxacin, an antimicrobial targeting bacterial DNA gyrase and topoisomerase IV, was classified as non-DDI. This reflects the specificity of norfloxacin to cause DNA damage only in bacterial cells. In Class 2B, three out of five antimetabolites, 6-mercaptopurine (6-MP), azidothymidine (AZT), and 5-azacytidine (5-AzaC), were classified as non-DDI. Unlike 5-Fluorouracil and Thioguanine, which incorporates into DNA and blocks DNA synthesis, AZT, an antiretroviral medication, interferes with reverse transcriptase; 5-AzaC inhibits DNA methylation; and 6-MP competes with the purine derivatives for the enzyme HGPRT and affects purine nucleotide synthesis by inhibiting phosphoribosyl pyrophosphate amidotransferase. The inhibition of reverse transcription and epigenetic changes do not cause typical genotoxic damage. 6-MP may eventually lead to genotoxicity, but the effects may not be evident until later time points.

Taken together, this validation study demonstrates that the TGx-DDI biomarker has utility for distinguishing DDI from non-DDI agents (Figure 3). Importantly, the biomarker is capable of differentiating compounds with irrelevant findings in CD assays from true DNA damaging agents; i.e., de-risking agents that are clastogenic *in vitro* but not *in vivo*. While all 12 agents in class 5 have been reported to be positive for CD assays, only one was positive using the TGx-DDI approach. Amongst the class 5 compounds, 90% were negative in the TGx-DDI.

### Technology and Cell System Compatibility of the TGx-DDI Biomarker

#### (1) Robustness of TGx-DDI on Various Technical Platforms

At the biomarker identification stage, global (i.e., genome-wide) transcriptomic profiling approaches such as gene expression microarrays and RNA-Seq are preferable technologies to comprehensively evaluate responsive genes. However, now that the TGx-DDI biomarker has been defined and validated, other methods that specifically query a targeted set of transcripts may be more desirable gene expression technologies for implementation, since they provide a simplified and higher throughput format (e.g., qRT-PCR, nCounter, and TempO-Seq platforms). Comparison of TGx-DDI biomarker performance across these technologies is shown in Table 3. These technologies are necessary for future expansion of the use of the TGx-DDI biomarker, or for its use in high throughput screening. Therefore, in subsequent studies the performance of TGx-DDI to differentiate DDI and non-DDI chemicals was evaluated using these methods (Li et al., 2017; Cho et al., 2019).

**Table 3:** Comparison of the multiplex gene expression measurement technologies.

**Features**	**Profiling**			**Targeted**	
	**Microarray**	**RNA-Seq**	**qRT-PCR**	**TempO-seq**	**nCounter^®^**
Sensitivity	Medium	Medium/ High	High	High	High
Need amplification and/or library	Yes	Yes	Yes	No	No
High-throughput	No	No	No	Yes	Yes
Automate	No	No	No	Yes	Yes
Cost of reagents	High	High	Medium	Medium	Medium
Cost of labor	High	High	Medium	Medium	Low
Readout	Analog	Digital	Analog	Digital	Digital

An alternative and widely used analytical platform in most molecular biology laboratories today is qRT-PCR. qRT-PCR is practical for TGx-DDI analysis and is a standard method for validation of transcriptional changes. Cho et al. developed a Taqman qRT-PCR version of TGx-DDI biomarker and showed comparable results with microarray data (Cho et al., 2019). In this study, 27 of the 28 model agents used in learning set, and 21 of the 24 external validation chemicals were accurately classified.

As the TGx-DDI biomarker consists of multiple genes, methods compatible for multiplex gene expression measurement are required. As shown in Table 3, most of these methods, such as microarrays, RNA-Seq, and multiplex qRT-PCR, are not high throughput, therefore, not feasible for the large-scaled genotoxicity screening. Low throughput is also one of the limitations of the standard genotoxicity assays. To meet the need for high-throughput methods for genotoxicity assessment, bridging studies of TGx-DDI on nCounter®, a direct digital counting technology that measures expression of up to 800 genes simultaneously was carried out. The technical robustness and reproducibility of TGx-DDI on nCounter® system was evaluated (Li et al., 2017). Results derived from nCounter® system were highly consistent with previous microarray data. A correlation of the log2-fold-changes of the transcripts in TGx-DDI as measured by nCounter® system vs. microarrays yielded linear fits with correlation coefficients of 0.91 (Li et al., 2017). All 28 model agents used in the learning set were correctly classified. Classification results of chemicals in the five classes of the validation study by nCounter® showed the equivalent accuracy to microarrays in terms of derisking the agents with irrelevant positive CD results, and higher sensitivity than microarray for detecting weaker DDI chemicals (Li et al., 2017). The throughput of each TGx-DDI nCounter® assay reaches 96 samples and can be used for direct cell lysate hybridization, which facilitates the incorporation of an automated assay procedure. Thus, this method provides a high-throughput approach that is compatible with automation, and promises to enable large-scale genotoxicity screening if needed.

In addition to the standalone TGx-DDI assays, this biomarker has also been integrated into the S1500-plus gene set for the high-throughput transcriptomics project that is part of the NIEHS' Tox21 consortium (Mav et al., 2018). It is expected that with these rapidly accumulating data, the specificity and sensitivity of this transcriptomic biomarker and its value in de-risking irrelevant positive CD results will be fully validated.

#### (2) Compatibility With Different Cell Systems

Despite the fact that most TGx-DDI studies thus far have used cultured TK6 cells, it is expected that transcriptional regulation in response to DDI agents should be similar in various human cells with intact DNA damage response pathways. This theoretical understanding supports the idea that the uses of the TGx-DDI biomarker are not limited to one cell system. TK6, a lymphoblastoid cell line, is not metabolically competent. Using TK6 in the presence of rat liver S9 microsomal fraction enabled robust DDI classification for those chemicals requiring metabolic activation by TGx-DDI (Buick et al., 2015; Corton et al., 2018). In addition, studies showed that TGx-DDI successfully classified DDI and non-DDI compounds in a gene expression data set from cultured HepaRG cells treated with a variety of compounds (Buick et al., 2015). Since HepaRG cells are derived from human hepatocytes and maintain drug metabolizing activity, the use of these cells for TGx-DDI testing might eliminate the need for metabolic activation using S9 microsomal fraction that is needed in case of TK6 cells.

#### (3) Bridging Studies for New Analytical Platforms and Cell Systems

With rapid innovation of transcriptomic technologies, the compatibility of the biomarker with new platforms and/or cell system is critical in broadening its long-term uses. To test whether a new platform or system is adaptable, bridging studies are needed to first compare its performance in DDI classification against the established methods. When incorporating a new method or cell system in the TGx-DDI assay, a bridging study on 28 agents used in the learning set is recommended. Optimal filtering, normalization and analysis parameters/approaches should be established. QA/QC metrics of the platform along with the recommended positive and negative controls should be applied. Performance should be tested against the published data using the SOP developed for the new system.

### Utility of TGx-DDI in Risk Assessment

**Figure 4:**
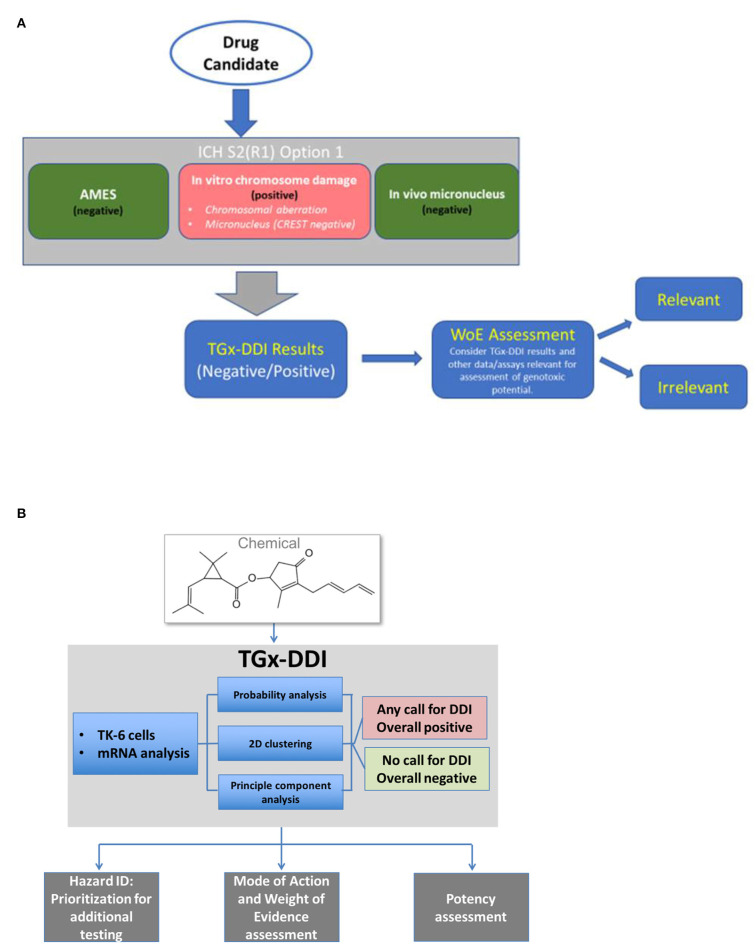
Proposed workflow for applying the TGx-DDI biomarker for genotoxicity assessment of **(A)** candidates in pharmaceutical drug development or of **(B)** industrial and environmental chemicals.

The potential to use TGx-DDI to inform hazard identification and risk assessment depends on the regulatory question or “context of use.” In this respect, the contexts of use for pharmaceuticals in drug development differ slightly from assessment of environmental and agricultural chemicals (Li et al., 2017) (Figure 4). To facilitate the application of this panel in decision-making, a consortium of scientists established within the Health and Environmental Sciences Institute (HESI) submitted the TGx-DDI biomarker to the US FDA's formal regulatory “qualification” program. HESI is a non-profit organization with the mission to engage scientists from academia, government, industry, research, and non-governmental organizations to identify and resolve global health and environmental issues. The HESI eSTAR (Emerging Systems Toxicology for Assessment of Risk) Committee is dedicated to developing innovative systems toxicology approaches for risk assessment. TGx-DDI biomarker development benefited from continuous feedback from regulators at the FDA. Initially the biomarker was reviewed by the agency within the Voluntary Genomic Data Submission program (Goodsaid et al., 2010). The comprehensive validation study, including selection of the 45 test chemicals (discussed in “Validation of the biomarker”), was designed based on feedback and guidance from the FDA. Following completion of this experiment, the TGx-DDI biomarker formally entered the Biomarker Qualification Program at the FDA. The biomarker qualification process requires periodical review of qualification plans and feedback from the agency. For example, the team recently reached an agreement with the FDA on the “context of use” statement for pharmaceutical applications. As of publication, the biomarker is still under regulatory review although a letter of support for the biomarker was issued by the FDA in October 2017. Meanwhile, HESI eSTAR Committee is expanding the reach to other regions by initiating interactions with European EMA and Japanese PMDA.

In addition to formal regulatory qualification, a complementary approach to facilitate and accelerate the implementation of new methods is through the conduct of case studies to demonstrate application. Below we describe different contexts of use for pharmaceuticals and chemicals assessment, and provide case study examples of application, including both qualitative and quantitative applications.

## Application 1: Use of TGx-DDI as a de-Risking Tool in Drug Development

The TGx-DDI biomarker has been proposed as a tool for assessing the relevance of positive findings in the *in vitro* CD assays (Goodsaid et al., 2010; Li et al., 2017; Cho et al., 2019). As described above, the high incidence of positive findings for *in vitro* CD assays that is irrelevant to human health provides a challenge to pharmaceutical industry and regulatory agencies.

To de-risk positive findings, the TGx-DDI biomarker should be applied as a follow-up to a positive result in an *in vitro* CD assay in mammalian cells (ICH S2(R1) option 1 battery) for a compound otherwise negative in mutation assays. A positive TGx-DDI result provides mechanistic information to indicate that the compound causes DNA damage that is potentially relevant to the manifestation of *in vivo* mutagenicity and carcinogenicity. For compounds classified as irrelevant using the TGx-DDI approach, this mechanistic information can be used as a rationale to reduce or eliminate the need for additional animal testing for genotoxicity beyond the required rodent *in vivo* micronucleus tests.

### Case Example

A case study on the use of the TGx-DDI biomarker in de-risking positive CD results for this context of use is provided in Li et al. (2015). In this example, three compounds were selected for analysis: isopropyl methanesulfonate (iPMS), 3-nitropropionic acid (3-NP), and tri-methylxanthine (caffeine). iPMS is a potent alkylating agent and a positive control DDI agent. 3-NP is a non-DDI agent that is toxic because it irreversibly inhibits succinate dehydrogenase, a critical enzyme involved in the citric acid cycle and electric transport chain, and thus causes severe energy impairment. 3-NP has previously yielded positive Ames assay data that were thought to be the result of compound contamination (Zeiger et al., 1988). Caffeine is negative in the Ames bacterial mutagenicity assay but gives positive *in vitro* CD test results that are not reproducible *in vivo* (Goodsaid et al., 2010). Analysis using the TGx-DDI biomarker in TK6 cells by DNA microarrays led to a positive call for iPMS and negative calls for 3-NP and caffeine as predicted based on their mechanisms of action. A Salmonella mutation assay was conducted to confirm the lack of genotoxicity of 3-NP, further supporting that this chemical does not cause genotoxicity. Caffeine is well-known to cause *in vitro* CD (Weinstein et al., 1975) through confounding cytotoxicity. The negative TGx-DDI call, in an example like this, indicates that caffeine's positive CD findings are not relevant to human cancer risk.

## Application 2: Use of TGx-DDI for Compound Screening and Prioritization in Drug Development and Chemicals Assessment

(1) With the availability and ease of use of high-throughput transcriptomic technologies (e.g., NanoString and BioSpyder technologies), the TGx-DDI biomarker can readily be applied for genetic toxicology hazard identification of lead compounds in drug development and of industrial and environmental chemicals.

A promising application of the TGx-DDI biomarker in the drug development pipeline is in the identification and prioritization of compounds that do not cause DNA damage. Here, the TGx-DDI could be used as a high-throughput approach for genotoxicity assessment in early compound screening, together with other novel high-throughput genotoxicity testing tools (e.g., the high-throughput comet assay).

The primary area of interest in chemicals assessment is in rapid genotoxicity hazard identification for data-poor chemicals that are present in the environment and/or commerce. International regulatory agencies are currently faced with the challenge of evaluating thousands of chemicals that lack conventional toxicity testing data. High-throughput transcriptomics has been evaluated by a variety of scientists as a tool to evaluate a very broad array of biological endpoints in a single experiment. Thus, the use of predictive biomarkers, such as TGx-DDI, to efficiently extract meaningful hazard information from transcriptomic signatures is a clear need in this area. As described above for pharmaceuticals, a DDI classification by TGx-DDI from an *in vitro* assessment of relevant human cells (i.e., possessing an intact p53-signaling response) could be used to identify agents as genotoxic and prioritize these chemicals for further testing. In fact, TGx-DDI might also have utility in retrospective application to existing gene expression data sets for this purpose.

### Case Example

Corton et al. demonstrated how the TGx-DDI biomarker could be used to rapidly screen large compendia of transcriptional profiles to identify toxicants that are potentially DDI (Corton et al., 2018). In this example, expression profiles from a commercially available gene expression database called BaseSpace Correlation Engine (BSCE) (https://www.illumina.com/products/by-type/informatics-products/basespace-correlation-engine.html) were analyzed. The correlation of the transcriptional profiles of human cells in culture (ratios of controls vs. chemically exposed) and the average fold changes of the genes in the TGx-DDI biomarker were evaluated using the Running Fisher algorithm (Kupershmidt et al., 2010). This method examines the overlap in the alteration of genes between the biomarker and each transcriptional profile in BSCE to identify significant positive (or negative) correlations. The approach accurately identified TK6 cells exposed to DDI and non-DDI agents from the original studies (Buick et al., 2015; Li et al., 2015, 2017; Yauk et al., 2016) with balanced accuracies of 87–97%, depending on the threshold for determining DDI positives. In addition, DDI agents in the metabolically competent hepatocyte cell line HepaRG (15 chemicals: five true positives and 10 true negatives) were also accurately identified [accuracy = 90%; there was one false negative (2-nitrofluorene) and no false positives]. Overall, these results demonstrate the utility of the biomarker to rapidly and accurately screen expression profiles from large numbers of chemicals to identify potential DDI agents.

(2) Weight of evidence analysis: The TGx-DDI biomarker can also be used in parallel with conventional *in vitro* genotoxicity tests to provide weight of evidence in hazard assessment. A DDI classification by TGx-DDI supports that the agent under study has the ability to cause DNA damage that is relevant to humans and *in vivo* genotoxicity.

### Case Example

In 2015, Moffat et al. undertook a case study to evaluate the use of toxicogenomics in the risk assessment of benzo[a]pyrene (BaP), a well-studied carcinogen (Moffat et al., 2015). The work aimed to explore the concordance of risk assessment outcomes for BaP using conventional vs. toxicogenomic approaches. Within their case study, the team of investigators used data from TGx-DDI TK6 cell analyses done in conjunction with micronucleus testing (in the presence of rat liver S9) to demonstrate a concentration-dependent increase in cytotoxicity, micronucleus formation, upregulation of known BaP-induced response genes, and induction of TGx-DDI biomarker genes. The author concluded that this analysis supported that DNA damage is a human-relevant key event in BaP's mode of action for carcinogenicity. Indeed, evidence demonstrating that expression profiles of BaP-treated TK6 cells clustered with those of other DNA-reactive genotoxic agents in the TGx-DDI database provided compelling evidence of its genotoxic potential. Overall, TGx-DDI results in human cell cultures were used in parallel with *in vivo* rodent studies to demonstrate how such data can be used as weight of evidence that an agent is a genotoxic carcinogen in Health Canada's risk assessments.

(3) Potency assessment: Finally, the response of the biomarker genes can also be used to determine a chemical or drug's genotoxic potency for quantifying potential hazard and for further prioritization. The field of genotoxicity testing is currently transitioning from purely qualitative (yes/no) classifications to quantitative approaches to assess the dose at which adverse genotoxic effects occur (White and Johnson, 2016; Wills et al., 2016a,b). The most frequently used approach involves benchmark dose (BMD) modeling of the transcriptional response of individual genes to identify the concentration/dose at which a predefined increase above background occurs (e.g., 10% increase is a standard benchmark response). Median BMDs are then generated for gene sets. Such quantitative approaches have been proposed by the US National Toxicology Program (NTP) to establish the dose at which biological effects begin to occur (National Toxicology Program, 2018). An obvious use of genomic BMDs is in the derivation of *in vitro* potency rankings of chemical agents. In this application, TGx-DDI biomarker genes for chemicals identified as genotoxic hazards based on the classification approach are subject to BMD modeling. The median gene BMD is then used as a potency estimate. This potency estimate can be compared to prototype toxicant BMDs for chemical prioritization for further *in vivo* genotoxicity or cancer testing. Such an approach could be further paired with *in vitro*-*in vivo* extrapolation to compare the human administered daily equivalent of the gene set BMD and the known human exposure.

### Case Example

Buick et al. used the TGx-DDI biomarker in parallel with the *in vitro* micronucleus test to evaluate two chemicals of regulatory interest at Health Canada: disperse orange (DO: an orange azo dye 3-[[4-[(4-Nitrophenyl)azo]phenyl] benzylamino]propanenitrile) and 1,2,4-benzenetriol (BT) (Buick et al., 2017). BT caused a robust increase in micronucleus frequency and concordant declines in relative survival. In contrast, DO exhibited weak induction of micronuclei that was <2-fold above controls at the highest concentration. The TGx-DDI biomarker was concordant with these results, providing supporting weight of evidence that both chemicals are DDI, despite the weak response for DO. BMD modeling was then applied to explore the concentration at which TGx-DDI genes were altered and BMDs were compared to those derived from similar experiments on the prototype genotoxic carcinogen BaP. Potency rankings of the chemicals were identical for the micronucleus test and TGx-DDI biomarker, supporting the use of the TGx-DDI gene set in this application. The results indicated that BaP was the most potent chemical, followed by BT and DO. In addition, the results revealed comparable BMDs for the micronucleus frequency and TGx-DDI gene responses with a 10% benchmark response (potencies within 10x). Overall, the work demonstrates how BMD modeling of the TGx-DDI biomarker can be used to evaluate and compare the potency of pharmaceuticals and chemicals.

## Summary

Use of the TGx-DDI transcriptomic biomarker is intended to provide high specificity and additional mechanistic information to augment current genotoxicity hazard assessment. This information can be useful for de-risking compounds with isolated *in vitro* positive CD findings. For this application, it should be applied following positive *in vitro* mammalian cell CD assays such as the *in vitro* MN assay, with negative CREST (antikinetochore) staining. In addition, application of the biomarker as a high-throughput transcriptomic screening approach for genotoxic hazard identification (Li et al., 2017) may lower expense in new drug/compound development and reduce animal use in safety testing. Such an approach also has clear value for application in screening of data poor chemicals to identify potential hazards, whereby mode of action-specific signatures can be applied in parallel to identify a variety of different potential hazards. TGx-DDI is the first transcriptomic biomarker that has been extensively validated using different cell systems and on various technical platforms.

Although surmountable, the development and application of any biomarker can have challenges. For example, as platforms and technologies change approaches to their validation must be similarly fluid and responsive. Such approaches will ideally optimize prior experience and data by focusing on performance-based outcomes that can be applied across different technical platforms rather than focusing on literal 1:1 correlation. The use of well-designed bridging studies—as described here—can provide a ready mechanism for achieving this objective. Other variables, such as temporal differences in the biological response to different chemical agents, can confound interpretation if not appropriately assessed. The use of time-series studies are recommended to optimize study design and interpretation of the biomarker results.

Overall, the strategies and methods that were used in identifying this biomarker can serve as a prototype in toxicogenomics for developing transcriptomic biomarkers for other types of toxicity.

## Author Contributions

H-HL, CY, JA, and AF made substantial contributions in reviewing literatures, organizing the manuscript, and drafting the paper. RC, DH, AW, RF, HE-Z, and SP made critical edits.

### Conflict of Interest

HE-Z is employed by company Bayer AG. RC was employed by company Amelia Technologies LLC. All other authors declare no competing interests.
